# Supplementary material for: O-GlcNAcylation regulates neurofilament-light assembly and function and is perturbed by Charcot-Marie-Tooth disease mutations
Source: Nat Commun. 2023 Oct 17;14:6558. doi: 10.1038/s41467-023-42227-0 (PMC10582078; doi:10.1038/s41467-023-42227-0)
Supplement: Supplementary file 1 — Supplementary Information [file 41467_2023_42227_MOESM1_ESM.pdf]

O-GlcNAcylation regulates neurofilament-light assembly and function and is perturbed by Charcot-Marie-Tooth disease mutations

Duc T. Huynh[1], Kalina N. Tsolova[1], Abigail J. Watson[1], Sai Kwan Khal[1], Jordan R. Green[2], Di Li[1], Jimin Hu[1], Erik J. Soderblom[3], Jen-Tsan Chi[4], Chantell S. Evans[2] and Michael Boyce[1,2*]

1. Department of Biochemistry, 2. Department of Cell Biology, 3. Proteomics and Metabolomics Shared Resource, and 4. Department of Molecular Genetics and Microbiology, Duke University School of Medicine, Durham, NC 27710, USA
* Correspondence: michael.boyce@duke.edu

Supplementary Information

Supplementary Figure 1
Supplementary Figure 2
Supplementary Figure 3
Supplementary Figure 4
Antibody validation
Supplementary Table 1
Supplementary Table 2
Supplementary Table 3

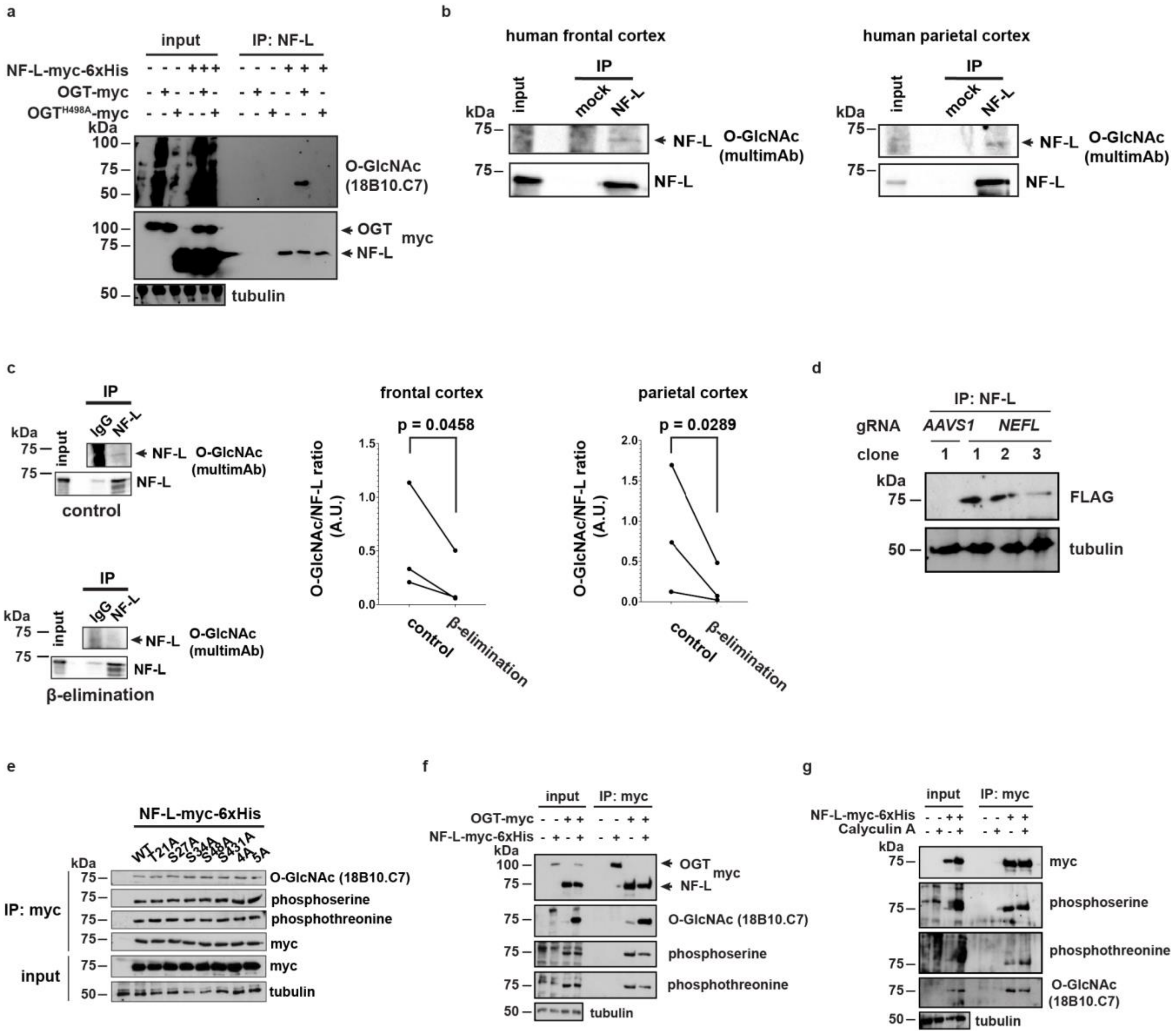

Supplementary Figure 1: Site-specific O-GlcNAcylation of the human NF-L head and tail domains. a 293T cells were transfected with NF-L-myc-6xHis ± OGT-myc or OGT^{H498A}-myc for 24 hrs and lysates were analyzed by NF-L IP and IB (n=3 biological replicates). **b** Human frontal or parietal cortex homogenates were analyzed by NF-L IP and IB. **c** Human frontal or parietal cortex homogenates were analyzed by NF-L IP, on-blot β-elimination, and IB. Left: Representative frontal cortex IP/IBs. Right: Normalized O-GlcNAc signal (O-GlcNAc/NF-L ratio) was calculated. Data are shown as mean ± SEM and assessed by Student's two-tailed t-test (n=3 biological replicates). **d** Lysates from single cell-derived clones of endogenously tagged NF-L-3xFLAG-6xHis 293T cells or negative control were analyzed by NF-L IP and IB (n=2 biological replicates). **e** *NEFL*$^{-/-}$ 293T cells were transfected with WT or glycosite mutant NF-L-myc-6xHis for 24 hrs, and lysates were analyzed by myc IP and IB (n=3 biological replicates). **f** 293T cells were transfected with NF-L-myc-6xHis ± OGT-myc for 24 hrs and lysates were analyzed by NF-L IP and IB with phospho-specific antibodies (n=3 biological replicates). **g** 293T cells were transfected with NF-L-myc-6xHis for 24 hrs, treated with calyculin A (100 nM, 15 min), and analyzed by NF-L IP and IB with phospho-specific antibodies (n=2 biological replicates).

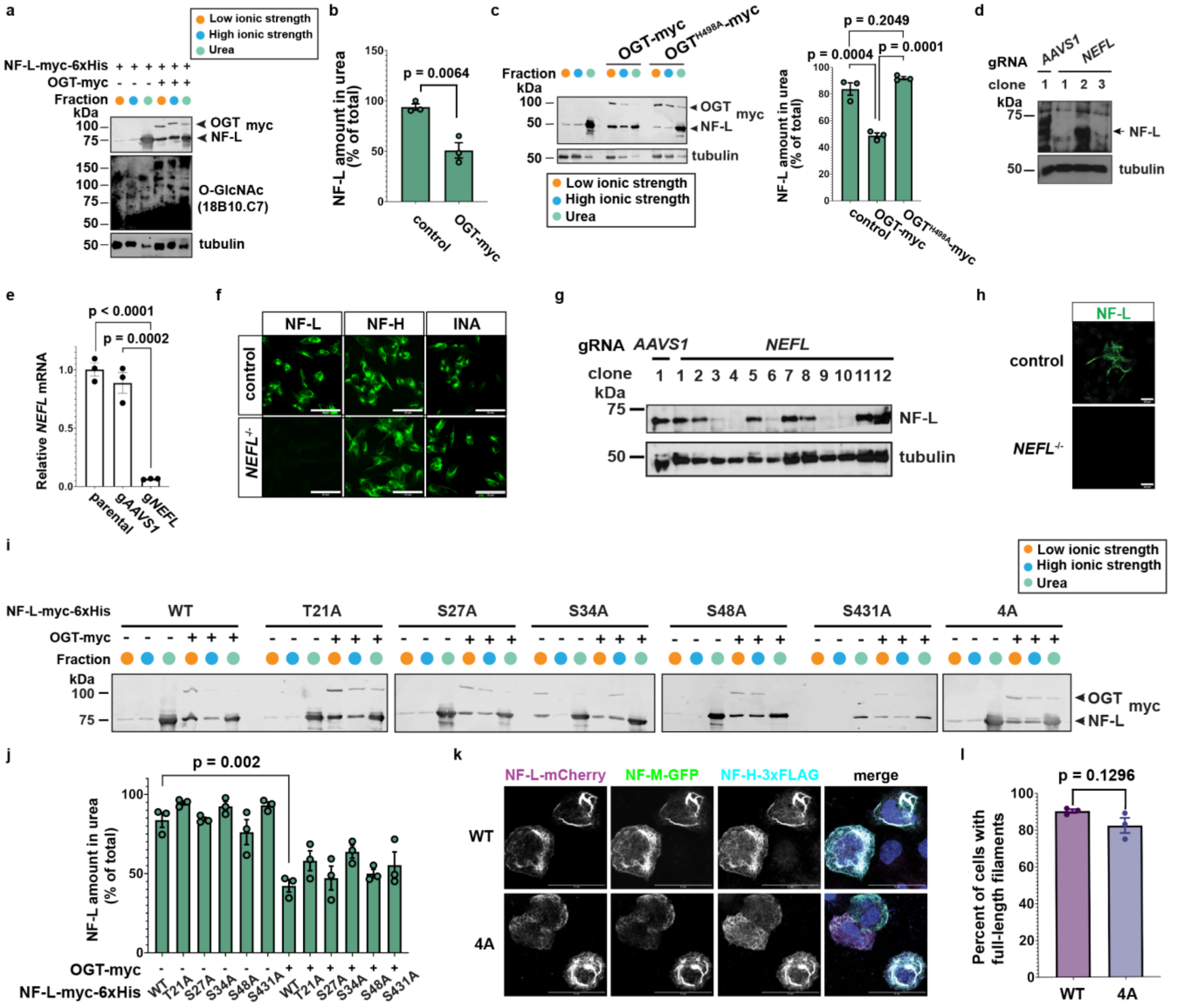

Supplementary Figure 2: NF-L O-GlcNAcylation influences NF-L assembly state and filament formation. a SH-SY5Y cells were transfected with NF-L-myc-6xHis ± OGT-myc for 24 hrs and analyzed by differential extraction and IB. **b** NF-L amount extracted into urea buffer was calculated as percent of total NF-L across three fractions from the experiment described in **a**. Data were shown as mean ± SEM and assessed by Student's two-tailed t-test (n=3 biological replicates). **c** Left: 293T cells were transfected with NF-L-myc-6xHis ± OGT-myc or OGT^{H498A}-myc for 24 hrs and analyzed by differential extraction and IB. Right: NF-L amount extracted into urea buffer was calculated as percent of total NF-L across three fractions. Data were shown as mean ± SEM and assessed by one-way ANOVA/Tukey's post-hoc correction (n=3 biological replicates). **d-f** CRISPR/Cas9-mediated deletion of *NEFL* in a single cell-derived SH-SY5Y clone was verified by IB (**d**), by actin-normalized qPCR (**e**), and by IFA (Scale bar: 50 μm) (**f**). Data from qPCR are shown as mean ± SEM and assessed by one-way ANOVA/Tukey's post-hoc correction (n=3 biological replicates). **g-h** CRISPR/Cas9-mediated deletion of *NEFL* in a single cell-derived 293T clone was verified by IB (**g**) and by IFA (Scale bar: 20 μm) (**h**). **i** *NEFL*$^{-/-}$ 293T cells were transfected with WT or glycosite mutant NF-L-myc-6xHis ± OGT-myc for 24 hrs and analyzed by differential extraction and IB. **j** NF-L amount

extracted into urea buffer was calculated as percent of total NF-L across three fractions from the experiment described in **i**. Data are shown as mean ± SEM and assessed by one-way ANOVA/Tukey's post-hoc correction (n=3 biological replicates). OGT co-expression significantly reduces the urea-extracted proportion of WT NF-L but not of any mutant. **k** SW13 vim- cells were transfected with WT or NF-L^{4A}-mCherry + NF-M-GFP + NF-H-3xFLAG (4:2:1 DNA ratio) for 24 hrs and analyzed by IFA. Scale bar: 50 μm. **l** Quantification of NF-L morphology in 100-170 cells per sample per biological replicate from the experiment described in **k**. Data are shown as mean ± SEM and assessed by Student's two-tailed t-test (n=3 biological replicates). ns: not significant.

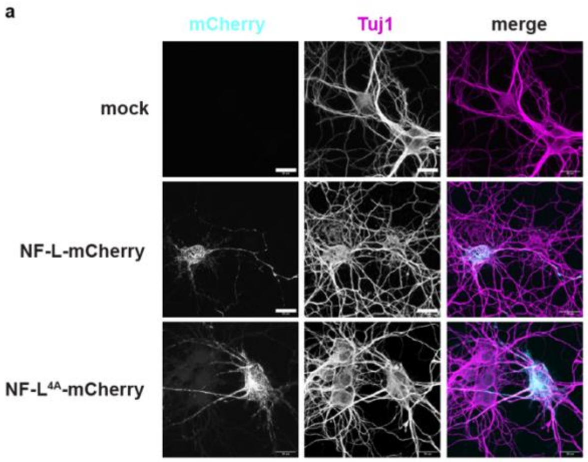

Supplementary Figure 3: NF-L expression does not disrupt the neuronal microtubule cytoskeleton. a Cultured E18 rat hippocampal neurons at day 6 *in vitro* were transfected with WT or NF-L^{4A}-mCherry for 24 hrs and analyzed by IFA with Tuj1 (n=3 biological replicates). Scale bar: 20 μm.

a

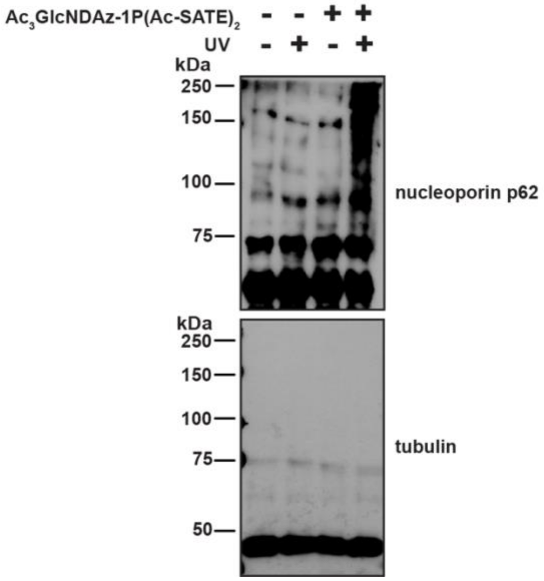

b

Top protein IDs with their spectral counts are shown			
Identified protein (Accession)	Molecular weight (kDa)	Control	+Ac_3GlcNDAz-1P(Ac-SATE)$_2$
Neurofilament light (NF-L)	62	854	1466
Keratin, type II cytoskeletal 2 epidermal (K22E)	65	638	422
Keratin, type II cytoskeletal 1 (K2C1)	66	465	409
Keratin, type I cytoskeletal 10 (K1C10)	59	457	407
Keratin, type I cytoskeletal 9 (K1C9)	62	357	303
Serum albumin (ALBU_BOVIN)	69	160	159
Myosin-9 (MYH9)	227	239	76
α–internexin (AINX)	55	0	180

Supplementary Figure 4: Direct, O-GlcNAc-mediated interactions between NF-L and INA. **a** 293T cells were treated with DMSO (vehicle) or 100 μM Ac_3GlcNDAz-1P(Ac-SATE)$_2$ for 48 hrs, subjected to UV crosslinking, and analyzed by IB. Heavily glycosylated nucleoporin p62 is a positive control, whereas unglycosylated tubulin is a negative control (n=3 biological replicates). **b** 293T cells were transfected with NF-L-myc-6xHis ± 100 μM Ac_3GlcNDAz-1P(Ac-SATE)$_2$ for 48 hrs and subjected to UV crosslinking. Lysates were analyzed by tandem myc IP/Ni-NTA purification, SDS-PAGE, and colloidal blue staining. High molecular weight crosslinked NF-L complexes from ± Ac_3GlcNDAz-1P(Ac-SATE)$_2$ samples were excised from the gels and analyzed by MS proteomics. Top protein IDs are shown. INA was identified from Ac_3GlcNDAz-1P(Ac-SATE)$_2$-treated cells, whereas no INA peptides were detected in the corresponding gel region from DMSO-treated cell samples. All IDs except NF-L and INA are common contaminants in proteomics experiments.

Antibody validation
All antibodies used in this study were obtained from and validated by commercial suppliers for immunoblotting and/or immunostaining. Validation information for each antibody is provided below.

myc (9E10, Biolegend, 626802):
https://www.biolegend.com/en-us/products/purified-anti-c-myc-antibody-2873?GroupID=GROUP26#:~:text=The%209E10%20monoclonal%20antibody%20recognizes,activity%20of%20the%20cloned%20sequence.

O-GlcNAc (18B10.C7, EMD Millipore, 05-1244):
https://www.thermofisher.com/antibody/product/O-linked-N-acetylglucosamine-O-GlcNAc-Antibody-cslone-18B10-C7-Monoclonal/MA1-038

O-GlcNAc (RL2, Biolegend, 677902): https://www.biolegend.com/fr-ch/products/purified-anti-o-glcnac-antibody-12021?GroupID=GROUP26

O-GlcNAc (multimAb, Cell Signaling Technology, 82332):
https://www.cellsignal.com/products/primary-antibodies/o-glcnac-multimab-rabbit-mab-mix/82332?_requestid=561913

Phosphoserine (Abcam, ab9332): https://www.abcam.com/products/primary-antibodies/phosphoserine-antibody-ab9332.html

Phosphothreonine (Abcam, ab9337): https://www.abcam.com/products/primary-antibodies/phosphothreonine-antibody-ab9337.html

NF-L (C28E10, Cell Signaling Technology, 2837):
https://www.cellsignal.com/products/primary-antibodies/neurofilament-l-c28e10-rabbit-mab/2837

FLAG (M-2, Sigma Aldrich, F1804):
https://www.sigmaaldrich.com/US/en/product/sigma/f1804?gclid=Cj0KCQjw9fqnBhDSARIsAHlcQYQX__VZW8UF0pg5mL0AAjK39zyyggXDbULJ8OHU0LzOEJ8dD2w0oiwaAh6sEALw_wcB

NF-M (2H3, Developmental Studies Hybridoma Bank, 2H3):
https://www.citeab.com/antibodies/149748-2h3-neurofilament-nf-m

NF200 (Sigma-Aldrich, N4142): https://www.sigmaaldrich.com/US/en/product/sigma/n4142

β-III tubulin (Tuj1, R&D Systems, MAB1195-SP):
https://www.rndsystems.com/products/neuron-specific-beta-iii-tubulin-antibody-tuj-1_mab1195?gclid=Cj0KCQjw9fqnBhDSARIsAHlcQYQ97fiRuV0_3BuVhy9_U386_amk73OyTv61J5K_yLQhAi3N5yDohagaAnnUEALw_wcB&gclsrc=aw.ds

α-tubulin (Sigma Aldrich, T6074): https://www.sigmaaldrich.com/US/en/product/sigma/t6074

V5 (ThermoFisher Scientific, R960-25): https://www.thermofisher.com/antibody/product/V5-Tag-Antibody-clone-SV5-Pk1-Monoclonal/R960-25

V5 (D3H8Q, Cell Signaling Technology, 13202): https://www.cellsignal.com/products/primary-antibodies/v5-tag-d3h8q-rabbit-mab/13202

INA (2E3, Novus Biologicals, NB300-140): https://www.novusbio.com/products/alpha-internexin-antibody-2e3_nb300-140

INA/NF66 (EnCor Biotechnology, RPCA-a-Int): https://encorbio.com/product/rpca-a-int/

Nucleoporin p62 (BD Biosciences, 610498): https://www.bdbiosciences.com/en-us/products/reagents/microscopy-imaging-reagents/immunofluorescence-reagents/purified-mouse-anti-nucleoporin-p62.610498

Goat HRP-conjugated anti-mouse IgG secondary antibody (SouthernBiotech, 1030-05): https://www.southernbiotech.com/goat-anti-mouse-igg-human-ads-hrp-1030-05

Goat HRP-conjugated anti-mouse kappa secondary antibody (SouthernBiotech, 1050-05): https://www.southernbiotech.com/goat-anti-mouse-kappa-hrp-1050-05

Goat HRP-conjugated anti-rabbit IgG secondary antibody (SouthernBiotech, 4030-05): https://www.southernbiotech.com/goat-anti-rabbit-igg-hrp-4030-05

Goat HRP-conjugated anti-rabbit light-chain secondary antibody (SouthernBiotech, 4060-05): https://www.southernbiotech.com/mouse-anti-rabbit-light-chain-hrp-sb62a-4060-05

Goat IRDye 800CW-conjugated anti-mouse IgG (H+L) secondary antibody (Li-Cor, 925-32210): https://www.licor.com/bio/reagents/irdye-800cw-goat-anti-mouse-igg-secondary-antibody

Goat IRDye 800CW-conjugated anti-rabbit IgG (H+L) secondary antibody (Li-Cor, 925-32211): https://www.licor.com/bio/reagents/irdye-800cw-goat-anti-rabbit-igg-secondary-antibody

Goat Alexa Fluor 488-conjugated anti-mouse (H + L) secondary antibody (Thermo Fisher Scientific, A-11001): https://www.thermofisher.com/antibody/product/Goat-anti-Mouse-IgG-H-L-Cross-Adsorbed-Secondary-Antibody-Polyclonal/A-11001

Goat Alexa Fluor 488-conjugated–anti-rabbit (H + L) conjugated secondary antibody (Thermo Fisher Scientific, A-11008): https://www.thermofisher.com/antibody/product/Goat-anti-Rabbit-IgG-H-L-Cross-Adsorbed-Secondary-Antibody-Polyclonal/A-11008

Goat Alexa Fluor 594-conjugated–anti-mouse (H + L) secondary antibody (Thermo Fisher Scientific, A-11005): https://www.thermofisher.com/antibody/product/Goat-anti-Mouse-IgG-H-L-Cross-Adsorbed-Secondary-Antibody-Polyclonal/A-11005

Goat Alexa Fluor 594-conjugated anti-rabbit (H + L) secondary antibody (Thermo Fisher Scientific, A-11012): https://www.thermofisher.com/antibody/product/Goat-anti-Rabbit-IgG-H-L-Cross-Adsorbed-Secondary-Antibody-Polyclonal/A-11012

Goat Alexa Fluor 647-conjugated anti-mouse IgG (H+L) cross-adsorbed secondary antibody (Thermo Fisher Scientific, A-21235): https://www.thermofisher.com/antibody/product/Goat-anti-Mouse-IgG-H-L-Cross-Adsorbed-Secondary-Antibody-Polyclonal/A-21235

Goat Alexa Fluor 647-conjugated anti-rabbit IgG (H+L) Highly cross-adsorbed secondary antibody (Invitrogen, A-32733): https://www.thermofisher.com/antibody/product/Goat-anti-Rabbit-IgG-H-L-Highly-Cross-Adsorbed-Secondary-Antibody-Polyclonal/A32733

Supplementary Table 1: Primer sequences

Mutation	Primer Sequences (F: Forward; R: Reverse)
P8L	F: 5' – CCTTCAGCTACGAGCTGTACTACTCGACCTC – 3'<br>R: 5' – GAGGTCGAGTAGTACAGCTCGTAGCTGAAGG
T21A	F: 5' – GCTACGTGGAGGCGCCCCGGGTG – 3'<br>R: 5' – CACCCGGGGCGCCTCCACGTAGC – 3'
P22R	F: 5' – CGTGGAGACGCGCCGGGTGCACA – 3'<br>R: 5' – TGTGCACCCGGCGCGTCTCCACG – 3'
P22S	F: 5' – GCTACGTGGAGACGAGCCGGGTGCACATCT – 3'<br>R: 5' – AGATGTGCACCCGGCTCGTCTCCACGTAGC – 3'
S27A	F: 5' – CGGGTGCACATCGCCAGCGTGCGCA – 3'<br>R: 5' – TGCGCACGCTGGCGATGTGCACCCG – 3'
S34A	F: 5' – GCGCAGCGGCTACGCCACCGCACGCTCA – 3'<br>R: 5' – TGAGCGTGCGGTGGCGTAGCCGCTGCGC – 3'
S48A	F: 5' – CGGCGCCGGTGGCTTCCTCGCTG – 3'<br>R: 5' – CAGCGAGGAAGCCACCGGCGCCG – 3'
L94P	F: 5' – GGAGAAGGCGCAGCCCCAGGACCTCAATG – 3'<br>R: 5' – CATTGAGGTCCTGGGGCTGCGCCTTCTCC – 3'
N98S	F: 5' – GCGCAGCTCCAGGACCTCAGTGACCGCTTC – 3'<br>R: 5' – GAAGCGGTCACTGAGGTCCTGGAGCTGCGC – 3'
A149V	F: 5' – ACCTGCGCCTGGTGGCGGAAGATGC – 3'<br>R: 5' – GCATCTTCCGCCACCAGGCGCAGGT – 3'
L268P	F: 5' – GCAGTACGAGAAGCCGGCCGCCAAGAACA – 3'<br>R: 5' – TGTTCTTGGCGGCCGGCTTCTCGTACTGC – 3'
Q332P	F: 5' – GCGCTGGAGAAGCCGCTGCAGGAGCTG – 3'<br>R: 5' – CAGCTCCTGCAGCGGCTTCTCCAGCGC – 3'
I384F	F: 5' – CGTGAAGATGGCTTTGGATTTTGAGATTGCAGCTTACA – 3'<br>R: 5' – TGTAAGCTGCAATCTCAAAATCCAAAGCCATCTTCACG – 3'
Y389C	F: 5' – GGATATTGAGATTGCAGCTTGCAGGAAACTCTTGGAAGG – 3'<br>R: 5' – CCTTCCAAGAGTTTCCTGCAAGCTGCAATCTCAATATCC – 3'
E396K	F: 5' – GAAACTCTTGGAAGGCAAGGAGACCCGACTCAG – 3'<br>R: 5' – CTGAGTCGGGTCTCCTTGCCTTCCAAGAGTTTC – 3'
S431A	F: 5' – CGGTTTACAGACCAGCGCCTATCTGATGTCCAC – 3'<br>R: 5' – GTGGACATCAGATAGGCGCTGGTCTGTAAACCG – 3'
P440L	F: 5' – CACCCGCTCCTTCCTGTCCTACTACACCA – 3'<br>R: 5' – TGGTGTAGTAGGACAGGAAGGAGCGGGTG – 3'
Y443N	F: 5' – CCTTCCCGTCCTACAACACCAGCCATGTC – 3'<br>R: 5' – GACATGGCTGGTGTTGTAGGACGGGAAGG – 3'
K467N	F: 5' – GCTGAGGAAGCCAATGATGAGCCCCCCTC – 3'<br>R: 5' – GAGGGGGGCTCATCATTGGCTTCCTCAGC – 3'
NEFL qPCR	F: 5' – ATGAGTTCCTTCAGCTACGAGC – 3'<br>R: 5' – CTGGGCATCAACGATCCAGA – 3'
b-actin qPCR	F: 5' – CACTCTTCCAGCCTTCCTTC – 3'<br>R: 5' – GGATGTCCACGTCACACTTC – 3'
NF-L^{4A}/^{5A}-myc-6xHis	Insert (NF-L^{4A} gene fragment):<br><br>Vector (WT or NF-L^{S431A}-myc-6xHis):

	F: 5’ – CGAGCTGCTGGTGCTGCGCCAGAAGCACTC – 3’<br>R: 5’ – GTAGCTGAAGGAACTCATGGTGGCGGATCCGAGCTC – 3’
NF-L-mCherry	Insert (NF-L-myc-6xHis):<br>F: 5' – AGCTCAAGCTTCGAATTCGCCACCATGAGTTCCTTCAGC – 3'<br>R: 5' – CGGGCCCGCGGTACCCCATCTTTCTTCTTAGCTGC – 3'<br><br>Vector (pmCherry vector, Takarabio, 632523):<br>F: 5' – GCAGCTAAGAAGAAAGATGGGGTACCGCGGGCCCG – 3'<br>R: 5' – GCTGAAGGAACTCATGGTGGCGAATTCGAAGCTTGAGCT – 3'
NF-M-GFP	Insert (phNFM, Addgene, 132597):<br>F: 5' – TACAAGTCCGGACTCAGATCTATGAGCTACACGTTGGAC – 3'<br>R: 5' –TATGATCAGTTATCTAGATCCGGTGTCACTCTGGGTGACTT – 3'<br><br>Vector (pEGFP-C1, Clontech):<br>F: 5' –GAAGTCACCCAGAGTGACACCGGATCTAGATAACTGATCATA – 3'<br>R: 5' – GTCCAACGTGTAGCTCATAGATCTGAGTCCGGACTTGTA – 3'
NF-H-3xFLAG	Insert (p3.1-NEFH, Addgene, 26978):<br>F: 5' – GACAAGCTTGCGGCCGCGAATATGATGAGCTTCGGC – 3'<br>R: 5' – TCACAGGGATGCCACCCGCTTCCCCTTGGCGGC – 3'<br><br>Vector (p3xFLAG-CMV-10, Sigma):<br>F: 5' – GCCGCCAAGGGGAAGCGGGTGGCATCCCTGTGA – 3'<br>R: 5' – GCCGAAGCTCATCATATTCGCGGCCGCAAGCTTGTC – 3'
OGT-3x-FLAG	Insert (OGT-myc-6xHis):<br>F: 5' – GACAAGCTTGCGGCCGCGAATATGGCGTCTTCCGTGGGC – 3'<br>R: 5' – TCACAGGGATGCCACCCGTGCTGACTCAGTGACTTC – 3'<br><br>Vector (p3xFLAG-CMV-10 vector, Sigma, E7658):<br>F: 5' – GAAGTCACTGAGTCAGCACGGGTGGCATCCCTGTGA – 3'<br>R: 5' – GCCCACGGAAGACGCCATATTCGCGGCCGCAAGCTTGTC – 3'
INA-V5	Insert (phINA, Addgene #132606):<br>F: 5' – GAGCTCGGATCCGCCACCATGAGCTTCGGCTCGGAG – 3'<br>R: 5' – CGGAGCCGCCGCCGCCTATTTTTTGGCTTGAAATGG – 3'<br><br>Vector (pcDNA4/V5-His A vector, ThermoFisher, V861-20):<br>F: 5' – CCATTTCAAGCCAAAAAATAGGCGGCGGCGGCTCCG – 3'<br>R: 5' – CTCCGAGCCGAAGCTCATGGTGGCGGATCCGAGCTC – 3'
UAP1^{F383G}-FLAG	Vector (UAP1^{F383G}-myc-6xHis):<br>F: 5'-GACTACAAAGACGATGACGACAAGTGAGTTTAAACCCGC-3'<br>R: 5'-CATCGTCTTTGTAGTCTCTAGAAATACCATTTTTCACCAGC-3'

Supplementary Table 2: Gene blocks and fragments

Gene sequence
>NF-L^{4A}<br>ATGAGTTCCTTCAGCTACGAGCCGTACTACTCGACCTCCTACAAGCGGCGCTACGTGG<br>AGGCGCCCCGGGTGCACATCGCCAGCGTGCGCAGCGGCTACGCCACCGCACGCTCA<br>GCTTACTCCAGCTACTCGGCGCCGGTGGCTTCCTCGCTGTCCGTGCGCCGCAGCTACT<br>CCTCCAGCTCTGGATCGTTGATGCCCAGTCTGGAGAACCTCGACCTGAGCCAGGTAGC<br>CGCCATCAGCAACGACCTCAAGTCCATCCGCACGCAGGAGAAGGCGCAGCTCCAGGA<br>CCTCAATGACCGCTTCGCCAGCTTCATCGAGCGCGTGCACGAGCTGGAGCAGCAGAAC<br>AAGGTCCTGGAAGCCGAGCTGCTGGTGCT<br><br>>*NEFL* homology arm (HA)-1<br>CAGGGAGCCATGGGATCCGCCGGCGACTACAAGGACCACGACGGCGATTATAAGGAT<br>CACGACATCGACTACAAAGACGACGATGACAAGAATATGCATACCGGTCATCATCACCA<br>TCACCATTTCGAAAAGTCA<br><br>> *NEFL* HA-2<br>GAGAATCTGTATTTTCAGGGAGCCATGGGATCCGCCGGCGAACAAAAACTCATCTCAG<br>AAGAGGATCTGAATATGCATACCGGTCATCATCACCATCACCATTTCGAAAAGTGATAA<br>CTCGAGTC

Supplementary Table 3: Antibody information

Antibody	Vendor and catalog number
myc (9E10)	Biolegend, 626802
O-GlcNAc (18B10.C7)	EMD Millipore, 05-1244
O-GlcNAc (RL2)	Biolegend, 677902
O-GlcNAc (multimAb)	Cell Signaling Technology, 82332
Phosphoserine	Abcam, ab9332
Phosphothreonine	Abcam, ab9337
NF-L (C28E10)	Cell Signaling Technology, 2837
FLAG (M-2)	Sigma Aldrich, F1804
NF-M	Developmental Studies Hybridoma Bank, 2H3
NF200 (N4142)	Sigma-Aldrich
β-III tubulin (Tuj1)	R&D Systems, MAB1195-SP
α-tubulin	Sigma Aldrich, T6074
V5	ThermoFisher Scientific, R960-25
V5 (D3H8Q)	Cell Signaling Technology, 13202
INA (2E3)	Novus Biologicals, NB300-140
INA/NF66	EnCor Biotechnology, RPCA-a-Int
Nucleoporin-p62	BD Biosciences, 610498
Goat HRP-conjugated anti-mouse IgG secondary antibody	SouthernBiotech, 1030-05
Goat HRP-conjugated anti-mouse kappa secondary antibody	SouthernBiotech, 1050-05
Goat HRP-conjugated anti-rabbit IgG secondary antibody	SouthernBiotech, 4030-05
Goat HRP-conjugated anti-rabbit light-chain secondary antibody	SouthernBiotech, 4060-05
Goat IRDye 800CW-conjugated anti-mouse IgG (H+L) secondary antibody	Li-Cor, 925-32210
Goat IRDye 800CW-conjugated anti-rabbit IgG (H+L) secondary antibody	Li-Cor, 925-32211
Goat Alexa Fluor 488-conjugated anti-mouse (H + L) secondary antibody	Thermo Fisher Scientific, A-11001
Goat Alexa Fluor 488-conjugated– anti-rabbit (H + L) conjugated secondary antibody	Thermo Fisher Scientific, A-11008
Goat Alexa Fluor 594-conjugated– anti-mouse (H + L) secondary antibody	Thermo Fisher Scientific, A-11005

Goat Alexa Fluor 594-conjugated anti-rabbit (H + L) secondary antibody	Thermo Fisher Scientific, A-11012
Goat Alexa Fluor 647-conjugated anti-mouse IgG (H+L) cross-adsorbed secondary antibody	Thermo Fisher Scientific, A-21235
Goat Alexa Fluor 647-conjugated anti-rabbit IgG (H+L) Highly cross-adsorbed secondary antibody	Invitrogen, A-32733
