# Supplementary material for: O-GlcNAcylation regulates neurofilament-light assembly and function and is perturbed by Charcot-Marie-Tooth disease mutations
Source: Nat Commun. 2023 Oct 17;14:6558. doi: 10.1038/s41467-023-42227-0 (PMC10582078; doi:10.1038/s41467-023-42227-0)
Supplement: Supplementary file 3 — Reporting Summary [file 41467_2023_42227_MOESM3_ESM.pdf]

nature portfolio

Corresponding author(s): Michael Boyce

Last updated by author(s): September 18, 2023

Reporting Summary

Nature Portfolio wishes to improve the reproducibility of the work that we publish. This form provides structure for consistency and transparency in reporting. For further information on Nature Portfolio policies, see our Editorial Policies and the Editorial Policy Checklist.

Please do not complete any field with "not applicable" or n/a. Refer to the help text for what text to use if an item is not relevant to your study.
For final submission: please carefully check your responses for accuracy; you will not be able to make changes later.

Statistics

For all statistical analyses, confirm that the following items are present in the figure legend, table legend, main text, or Methods section.

n/a	Confirmed	
☐	☒	The exact sample size (*n*) for each experimental group/condition, given as a discrete number and unit of measurement
☐	☒	A statement on whether measurements were taken from distinct samples or whether the same sample was measured repeatedly
☐	☒	The statistical test(s) used AND whether they are one- or two-sided<br>*Only common tests should be described solely by name; describe more complex techniques in the Methods section.*
☒	☐	A description of all covariates tested
☐	☒	A description of any assumptions or corrections, such as tests of normality and adjustment for multiple comparisons
☐	☒	A full description of the statistical parameters including central tendency (e.g. means) or other basic estimates (e.g. regression coefficient) AND variation (e.g. standard deviation) or associated estimates of uncertainty (e.g. confidence intervals)
☐	☒	For null hypothesis testing, the test statistic (e.g. *F*, *t*, *r*) with confidence intervals, effect sizes, degrees of freedom and *P* value noted<br>*Give P values as exact values whenever suitable.*
☒	☐	For Bayesian analysis, information on the choice of priors and Markov chain Monte Carlo settings
☒	☐	For hierarchical and complex designs, identification of the appropriate level for tests and full reporting of outcomes
☒	☐	Estimates of effect sizes (e.g. Cohen's *d*, Pearson's *r*), indicating how they were calculated

Our web collection on statistics for biologists contains articles on many of the points above.

Software and code

Policy information about availability of computer code

Data collection	Immunoblots were collected on LICOR Odyssey CLx; Immunofluorescence data on the inverted 780 single point scanning confocal microscope (Zeiss), and live-cell imaging on ELYRA7 (Zeiss)
Data analysis	GraphPad Prism (v.10.0.2), Imaris (v.10.0), LI-COR Odyssey CLx imaging software, Zeiss ELYRA7 imaging processing

For manuscripts utilizing custom algorithms or software that are central to the research but not yet described in published literature, software must be made available to editors and reviewers. We strongly encourage code deposition in a community repository (e.g. GitHub). See the Nature Portfolio guidelines for submitting code & software for further information

Data

Policy information about availability of data

All manuscripts must include a data availability statement. This statement should provide the following information, where applicable:

- Accession codes, unique identifiers, or web links for publicly available datasets
- A description of any restrictions on data availability
- For clinical datasets or third party data, please ensure that the statement adheres to our policy

The mass spectrometry proteomics data have been deposited to both MassIVE repository (Massive.ucsd.edu; User: MSV000091348_reviewer; Password: proteomics; Identifier: MSV000091348) and the ProteomeXchange Consortium via the PRIDE partner repository with the dataset identifier PXD045364 (Username: reviewer_pxd045364@ebi.ac.uk; Password: 638MKDmD)

Research involving human participants, their data, or biological material

Policy information about studies with human participants or human data. See also policy information about sex, gender (identity/presentation), and sexual orientation and race, ethnicity and racism.

Reporting on sex and gender	N/A
Reporting on race, ethnicity, or other socially relevant groupings	N/A
Population characteristics	N/A
Recruitment	N/A
Ethics oversight	N/A

Note that full information on the approval of the study protocol must also be provided in the manuscript.

Field-specific reporting

Please select the one below that is the best fit for your research. If you are not sure, read the appropriate sections before making your selection.

☒ Life sciences ☐ Behavioural & social sciences ☐ Ecological, evolutionary & environmental sciences

For a reference copy of the document with all sections, see nature.com/documents/nr-reporting-summary-flat.pdf

Life sciences study design

All studies must disclose on these points even when the disclosure is negative.

Sample size	For each experiment, the results were collected from three biological replicates to ensure consistent phenotypes and to perform statistical analyses
Data exclusions	No data were excluded
Replication	All experiments were repeated three times and the results were collected from three biological replicates
Randomization	No randomization was involved because this study does not involve human subjects or animals. However, reproducibility over three biological replicates and blinded quantification were performed to ensure scientific rigor
Blinding	Blinded quantification of immunofluorescence experiments was done wherever appropriate (i.e. an independent researcher quantifying the results was blinded to the experimental conditions)

Behavioural & social sciences study design

All studies must disclose on these points even when the disclosure is negative.

Study description	N/A
Research sample	N/A
Sampling strategy	N/A
Data collection	N/A
Timing	N/A
Data exclusions	N/A
Non-participation	N/A
Randomization	N/A

Ecological, evolutionary & environmental sciences study design

All studies must disclose on these points even when the disclosure is negative.

Study description	N/A
Research sample	N/A
Sampling strategy	N/A
Data collection	N/A
Timing and spatial scale	N/A
Data exclusions	N/A
Reproducibility	N/A
Randomization	N/A
Blinding	N/A

Did the study involve field work? ☐ Yes ☒ No

Field work, collection and transport

Field conditions	
Location	
Access & import/export	
Disturbance	

Reporting for specific materials, systems and methods

We require information from authors about some types of materials, experimental systems and methods used in many studies. Here, indicate whether each material, system or method listed is relevant to your study. If you are not sure if a list item applies to your research, read the appropriate section before selecting a response.

Materials & experimental systems

n/a	Involved in the study	
☐	☒	Antibodies
☐	☒	Eukaryotic cell lines
☒	☐	Palaeontology and archaeology
☐	☒	Animals and other organisms
☒	☐	Clinical data
☒	☐	Dual use research of concern
☒	☐	Plants

Methods

n/a	Involved in the study	
☒	☐	ChIP-seq
☒	☐	Flow cytometry
☒	☐	MRI-based neuroimaging

Antibodies

Antibodies used

Unless stated otherwise, primary antibodies are diluted 1:1,000 for immunoblotting and 1:400 for immunofluorescence; Goat HRP secondary antibodies 1:10,000; Goat IRDye 800CW secondary antibodies 1:30,000; Goat Alexa Fluor secondary antibodies 1:400.

Primary antibodies: myc (9E10, Biolegend, 626802), O-GlcNAc (18B10.C7, EMD Millipore, 05-1244), O-GlcNAc (RL2, Biolegend, 677902), O-GlcNAc (multimAb, Cell Signaling Technology, 82332), phosphoserine (Abcam, ab9332), phosphothreonine (Abcam, ab9337); NF-L (C28E10, Cell Signaling Technology, 2837), FLAG (M-2, Sigma Aldrich, F1804), NF-M (2H3, Developmental Studies Hybridoma Bank, 2H3), NF200 (Sigma-Aldrich, N4142), beta-III tubulin (Tuj1, R&D Systems, MAB1195-SP), alpha-tubulin (Sigma Aldrich, T6074, dilution 1:100,000), V5 (ThermoFisher Scientific, R960-25), V5 (D3H8Q, Cell Signaling Technology, 13202), INA (2E3, Novus Biologicals, NB300-140, dilution 1:2,000), INA/NF66 (EnCor Biotechnology, RPCA-a-Int), nucleoporin p62 (BD Biosciences, 610498)

Secondary antibodies: Goat HRP-conjugated anti-mouse IgG secondary antibody (SouthernBiotech, 1030-05), Goat HRP-conjugated anti-mouse kappa secondary antibody (SouthernBiotech, 1050-05), Goat HRP-conjugated anti-rabbit IgG secondary antibody (SouthernBiotech, 4030-05), Goat HRP-conjugated anti-rabbit light-chain secondary antibody (SouthernBiotech, 4060-05), Goat IRDye 800CW-conjugated anti-mouse IgG (H +L) secondary antibody (Li-Cor, 925-32210), Goat IRDye 800CW-conjugated anti-mouse anti-rabbit IgG (H+L) secondary antibody (Li-Cor, 925-32211), Goat Alexa Fluor 488-conjugated anti-mouse (H + L) secondary antibody (Thermo Fisher Scientific, A-11001), Goat Alexa Fluor 488-conjugated anti-rabbit (H + L) conjugated secondary antibody (Thermo Fisher Scientific, A-11008), Goat Alexa Fluor 594-conjugated anti-mouse (H + L) secondary antibody (Thermo Fisher Scientific, A-11005), Goat Alexa Fluor 594-conjugated anti-rabbit (H + L) secondary antibody (Thermo Fisher Scientific, A-11012), Goat Alexa Fluor 647-conjugated anti-mouse IgG (H+L) cross-adsorbed secondary antibody (Thermo Fisher Scientific, A-21235), Goat Alexa Fluor 647-conjugated anti-rabbit IgG (H+L) Highly cross-adsorbed secondary antibody (Invitrogen, A-32733)

Validation

All antibodies were validated by commercial suppliers for immunoblotting and/or immunofluorescence. Detailed information can be found on commercial websites.
myc: https://www.biolegend.com/en-us/products/purified-anti-c-myc-antibody-2873
O-GlcNAc (18B10.C7): https://www.thermofisher.com/antibody/product/O-linked-N-acetylglucosamine-O-GlcNAc-Antibody-clone-18B10-C7-Monoclonal/MA1-038
O-GlcNAc (RL2): https://www.biolegend.com/fr-ch/products/purified-anti-o-glcnac-antibody-12021?GroupID=GROUP26
O-GlcNAc (multimAb): https://www.cellsignal.com/products/primary-antibodies/o-glcnac-multimab-rabbit-mab-mix/82332?_requestid=561913
Phosphoserine: https://www.abcam.com/products/primary-antibodies/phosphoserine-antibody-ab9332.html
Phosphothreonine: https://www.abcam.com/products/primary-antibodies/phosphothreonine-antibody-ab9337.html
NF-L: https://www.cellsignal.com/products/primary-antibodies/neurofilament-l-c28e10-rabbit-mab/2837
FLAG: https://www.sigmaaldrich.com/US/en/product/sigma/f1804?gclid=Cj0KCQjw9fqnBhDSARIsAHlcQYQX__VZW8UF0pg5mL0AAjK39zyyggXDbULJ8OHU0LzOEJ8dD2w0oiwaAh6sEALw_wcB
NF-M: https://www.citeab.com/antibodies/149748-2h3-neurofilament-nf-m
NF200: https://www.sigmaaldrich.com/US/en/product/sigma/n4142
Tuj1: https://www.rndsystems.com/products/neuron-specific-beta-iii-tubulin-antibody-tuj-1_mab1195
alpha-tubulin: https://www.sigmaaldrich.com/US/en/product/sigma/t6074
V5 (R960-25): https://www.thermofisher.com/antibody/product/V5-Tag-Antibody-clone-SV5-Pk1-Monoclonal/R960-25
V5 (D3H8Q): https://www.cellsignal.com/products/primary-antibodies/v5-tag-d3h8q-rabbit-mab/13202
INA (2E3): https://www.novusbio.com/products/alpha-internexin-antibody-2e3_nb300-140
INA/NF66 (RPCA-a-Int): https://encorbio.com/product/rpca-a-int/
Nucleoporin p62: https://www.bdbiosciences.com/en-us/products/reagents/microscopy-imaging-reagents/immunofluorescence-reagents/purified-mouse-anti-nucleoporin-p62.610497
Goat HRP-conjugated anti-mouse IgG secondary antibody: https://www.southernbiotech.com/goat-anti-mouse-igg-human-ads-hrp-1030-05
Goat HRP-conjugated anti-mouse kappa secondary antibody: https://www.southernbiotech.com/goat-anti-mouse-kappa-hrp-1050-05
Goat HRP-conjugated anti-rabbit IgG secondary antibody: https://www.southernbiotech.com/goat-anti-rabbit-igg-hrp-4030-05
Goat HRP-conjugated anti-rabbit light-chain secondary antibody: https://www.southernbiotech.com/mouse-anti-rabbit-light-chain-hrp-sb62a-4060-05
Goat IRDye 800CW-conjugated anti-mouse IgG (H+L) secondary antibody: https://www.licor.com/bio/reagents/irdye-800cw-goat-anti-mouse-igg-secondary-antibody
Goat IRDye 800CW-conjugated anti-rabbit IgG (H+L) secondary: https://www.licor.com/bio/reagents/irdye-800cw-goat-anti-rabbit-igg-secondary-antibody
Goat Alexa Fluor 488-conjugated anti-mouse (H + L) secondary antibody: https://www.thermofisher.com/antibody/product/Goat-anti-Mouse-IgG-H-L-Cross-Adsorbed-Secondary-Antibody-Polyclonal/A-11001
Goat Alexa Fluor 488-conjugated–anti-rabbit (H + L) conjugated secondary antibody: https://www.thermofisher.com/antibody/product/Goat-anti-Rabbit-IgG-H-L-Cross-Adsorbed-Secondary-Antibody-Polyclonal/A-11008
Goat Alexa Fluor 594-conjugated–anti-mouse (H + L) secondary antibody: https://www.thermofisher.com/antibody/product/Goat-anti-Mouse-IgG-H-L-Cross-Adsorbed-Secondary-Antibody-Polyclonal/A-11005
Goat Alexa Fluor 594-conjugated anti-rabbit (H + L) secondary antibody: https://www.thermofisher.com/antibody/product/Goat-anti-Rabbit-IgG-H-L-Cross-Adsorbed-Secondary-Antibody-Polyclonal/A-11012
Goat Alexa Fluor 647-conjugated anti-mouse IgG (H+L) cross-adsorbed secondary antibody: https://www.thermofisher.com/antibody/product/Goat-anti-Mouse-IgG-H-L-Cross-Adsorbed-Secondary-Antibody-Polyclonal/A-21235
Goat Alexa Fluor 647-conjugated anti-rabbit IgG (H+L) Highly cross-adsorbed secondary antibody: https://www.thermofisher.com/antibody/product/Goat-anti-Rabbit-IgG-H-L-Highly-Cross-Adsorbed-Secondary-Antibody-Polyclonal/A32733

Eukaryotic cell lines

Policy information about cell lines and Sex and Gender in Research

Cell line source(s)

293T (ATCC CRL-11268), SH-SY5Y (ATCC CRL-2266), SW13 vim- (Snider lab), rat hippocampal neurons (Evans lab)

Authentication

Cell lines obtained from ATCC were authenticated by STR profiling. SW13 vim- cells were validated by the Snider lab by morphology, karyotyping, and immunofluorescence for the absence of vimentin (vim-). Rat hippocampal neurons were validated by the Evans lab for no mycoplasma or glial contaminatiion (via treatment with AraC)

Mycoplasma contamination

All cell lines are negative for mycoplasma testing

Commonly misidentified lines
(See ICLAC register)

No commonly misidentified lines were used in this study

Palaeontology and Archaeology

Specimen provenance

Specimen deposition

Dating methods

☐ Tick this box to confirm that the raw and calibrated dates are available in the paper or in Supplementary Information.

Ethics oversight

Note that full information on the approval of the study protocol must also be provided in the manuscript.

Animals and other research organisms

Policy information about studies involving animals; ARRIVE guidelines recommended for reporting animal research, and Sex and Gender in Research

Laboratory animals

For live-cell imaging, hippocampal primary neurons were dissected from embryonic day eighteen timed-pregnant CD Sprague-Dawley rats that were ordered from Charles River and housed after the delivery for 1-2 days by Duke's Division of Laboratory Animal Resources (DLAR)

Wild animals

This study did not involve wild animals

Reporting on sex

Neurons from male and female embryos were represented equally in this study

Field-collected samples

This study did not involve field-collected samples

Ethics oversight

All animal work in this study was performed under the oversight of the Duke University Institutional Animal Care and Use Committee, which reviewed and approved the written protocol (#A230-21-11) and regularly inspects all animal facilities. Rats were acquired through Duke’s DLAR, which has expert veterinary staff to attend to their daily care and well-being. Rats were housed in a Duke animal care facility accredited by the Association for Assessment and Accreditation of Laboratory Animal Care (AAALAC). All animal procedures, including euthanasia, were carried out according to the general guidelines of the US Animal Welfare Act and AAALAC and complied fully with all relevant ethical standards.

Note that full information on the approval of the study protocol must also be provided in the manuscript.

Clinical data

Policy information about clinical studies

All manuscripts should comply with the ICMJE guidelines for publication of clinical research and a completed CONSORT checklist must be included with all submissions.

Clinical trial registration	
Study protocol	
Data collection	
Outcomes	

Dual use research of concern

Policy information about dual use research of concern

Hazards

Could the accidental, deliberate or reckless misuse of agents or technologies generated in the work, or the application of information presented in the manuscript, pose a threat to:

No	Yes	
☒	☐	Public health
☒	☐	National security
☒	☐	Crops and/or livestock
☒	☐	Ecosystems
☒	☐	Any other significant area

Experiments of concern

Does the work involve any of these experiments of concern:

No	Yes	
☒	☐	Demonstrate how to render a vaccine ineffective
☒	☐	Confer resistance to therapeutically useful antibiotics or antiviral agents
☒	☐	Enhance the virulence of a pathogen or render a nonpathogen virulent
☒	☐	Increase transmissibility of a pathogen
☒	☐	Alter the host range of a pathogen
☒	☐	Enable evasion of diagnostic/detection modalities
☒	☐	Enable the weaponization of a biological agent or toxin
☒	☐	Any other potentially harmful combination of experiments and agents

Plants

Seed stocks	
Novel plant genotypes	
Authentication	

ChIP-seq

Data deposition

☐ Confirm that both raw and final processed data have been deposited in a public database such as GEO.

☐ Confirm that you have deposited or provided access to graph files (e.g. BED files) for the called peaks.

Data access links<br>*May remain private before publication.*	
Files in database submission	
Genome browser session<br>(e.g. UCSC)	

Methodology

Replicates	
Sequencing depth	
Antibodies	
Peak calling parameters	
Data quality	
Software	

Flow Cytometry

Plots

Confirm that:

☐ The axis labels state the marker and fluorochrome used (e.g. CD4-FITC).

☐ The axis scales are clearly visible. Include numbers along axes only for bottom left plot of group (a 'group' is an analysis of identical markers).

☐ All plots are contour plots with outliers or pseudocolor plots.

☐ A numerical value for number of cells or percentage (with statistics) is provided.

Methodology

Sample preparation	
Instrument	
Software	
Cell population abundance	
Gating strategy	

☐ Tick this box to confirm that a figure exemplifying the gating strategy is provided in the Supplementary Information.

Magnetic resonance imaging

Experimental design

Design type

Design specifications

Behavioral performance measures

Imaging type(s)

Field strength

Sequence & imaging parameters

Area of acquisition

Diffusion MRI ☐ Used ☐ Not used

Preprocessing

Preprocessing software

Normalization

Normalization template

Noise and artifact removal

Volume censoring

Statistical modeling & inference

Model type and settings

Effect(s) tested

Specify type of analysis: ☐ Whole brain ☐ ROI-based ☐ Both

Statistic type for inference

(See Eklund et al. 2016)

Correction

Models & analysis

n/a	Involved in the study
☐	☐ Functional and/or effective connectivity
☐	☐ Graph analysis
☐	☐ Multivariate modeling or predictive analysis

Functional and/or effective connectivity

Graph analysis

Multivariate modeling and predictive analysis

This checklist template is licensed under a Creative Commons Attribution 4.0 International License, which permits use, sharing, adaptation, distribution and reproduction in any medium or format, as long as you give appropriate credit to the original author(s) and the source, provide a link to the Creative Commons license, and indicate if changes were made. The images or other third party material in this article are included in the article's Creative Commons license, unless indicated otherwise in a credit line to the material. If material is not included in the article's Creative Commons license and your intended use is not permitted by statutory regulation or exceeds the permitted use, you will need to obtain permission directly from the copyright holder. To view a copy of this license, visit http://creativecommons.org/licenses/by/4.0/
