# Supplementary material for: O-GlcNAcylation regulates neurofilament-light assembly and function and is perturbed by Charcot-Marie-Tooth disease mutations
Source: Nat Commun. 2023 Oct 17;14:6558. doi: 10.1038/s41467-023-42227-0 (PMC10582078; doi:10.1038/s41467-023-42227-0)

Figure 1

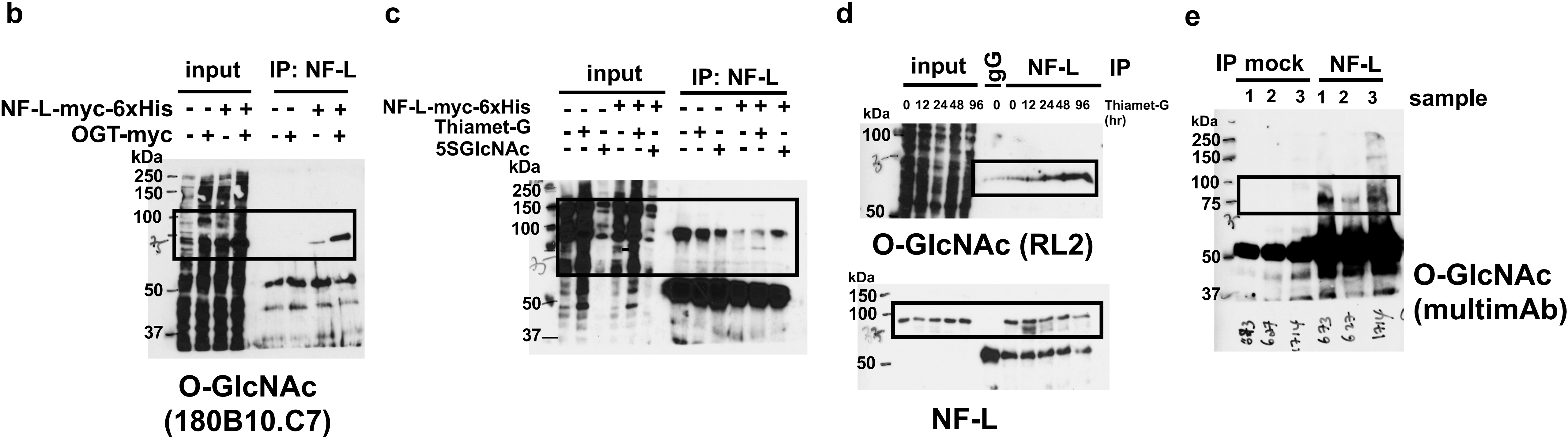
b
input
IP: NF-L
NF-L-myc-6xHis - - + + - - + +
OGT-myc - + - + - + - +
kDa
250
150
100
50
37
O-GlcNAc
(180B10.C7)
c
input
IP: NF-L
NF-L-myc-6xHis - - - + + + - - - + + +
Thiamet-G - + - - + - - + - - + -
5SGlcNAc - - + - - + - - + - - +
kDa
250
150
100
50
37
d
input
IgG
NF-L
IP
0 12 24 48 96 0 0 12 24 48 96
Thiamet-G (hr)
kDa
100
50
O-GlcNAc (RL2)
kDa
150
100
50
NF-L
e
IP mock
NF-L
1 2 3 1 2 3
sample
kDa
250
150
100
75
50
37
O-GlcNAc
(multimAb)

Figure 1

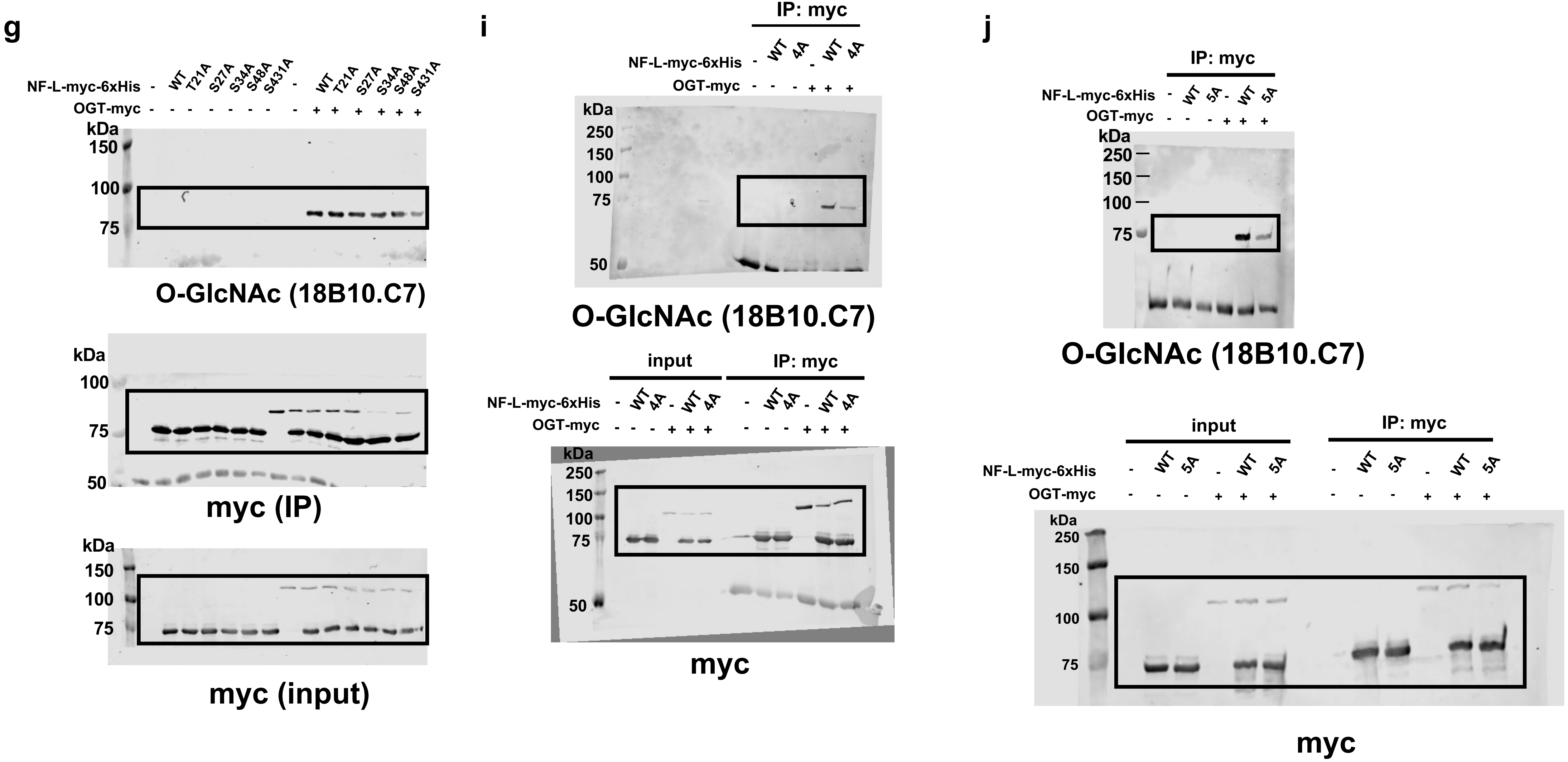

g
NF-L-myc-6xHis - WT T21A S27A S34A S48A S431A - WT T21A S27A S34A S48A S431A
OGT-myc - - - - - - - - + + + + + +
kDa
150
100
75
O-GlcNAc (18B10.C7)
kDa
100
75
50
myc (IP)
kDa
150
100
75
myc (input)
i
IP: myc
NF-L-myc-6xHis - WT 4A - WT 4A
OGT-myc - - - + + +
kDa
250
150
100
75
50
O-GlcNAc (18B10.C7)
input
IP: myc
NF-L-myc-6xHis - WT 4A - WT 4A - WT 4A - WT 4A
OGT-myc - - - + + + - - - + + +
kDa
250
150
100
75
50
myc
j
IP: myc
NF-L-myc-6xHis - WT 5A - WT 5A
OGT-myc - - - + + +
kDa
250
150
100
75
O-GlcNAc (18B10.C7)
input
IP: myc
NF-L-myc-6xHis - WT 5A - WT 5A - WT 5A - WT 5A
OGT-myc - - - + + + - - - + + +
kDa
250
150
100
75
myc

Figure 2

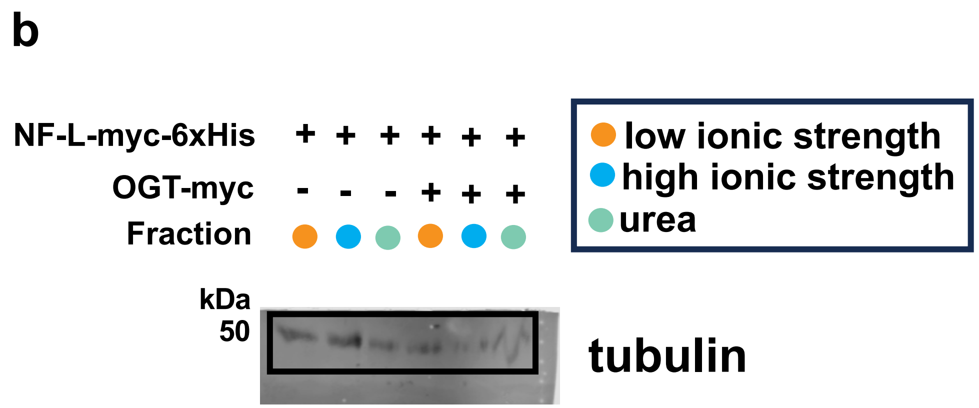
b
NF-L-myc-6xHis + + + + + +
OGT-myc - - - + + +
Fraction
kDa
50
tubulin
low ionic strength
high ionic strength
urea

Figure 4

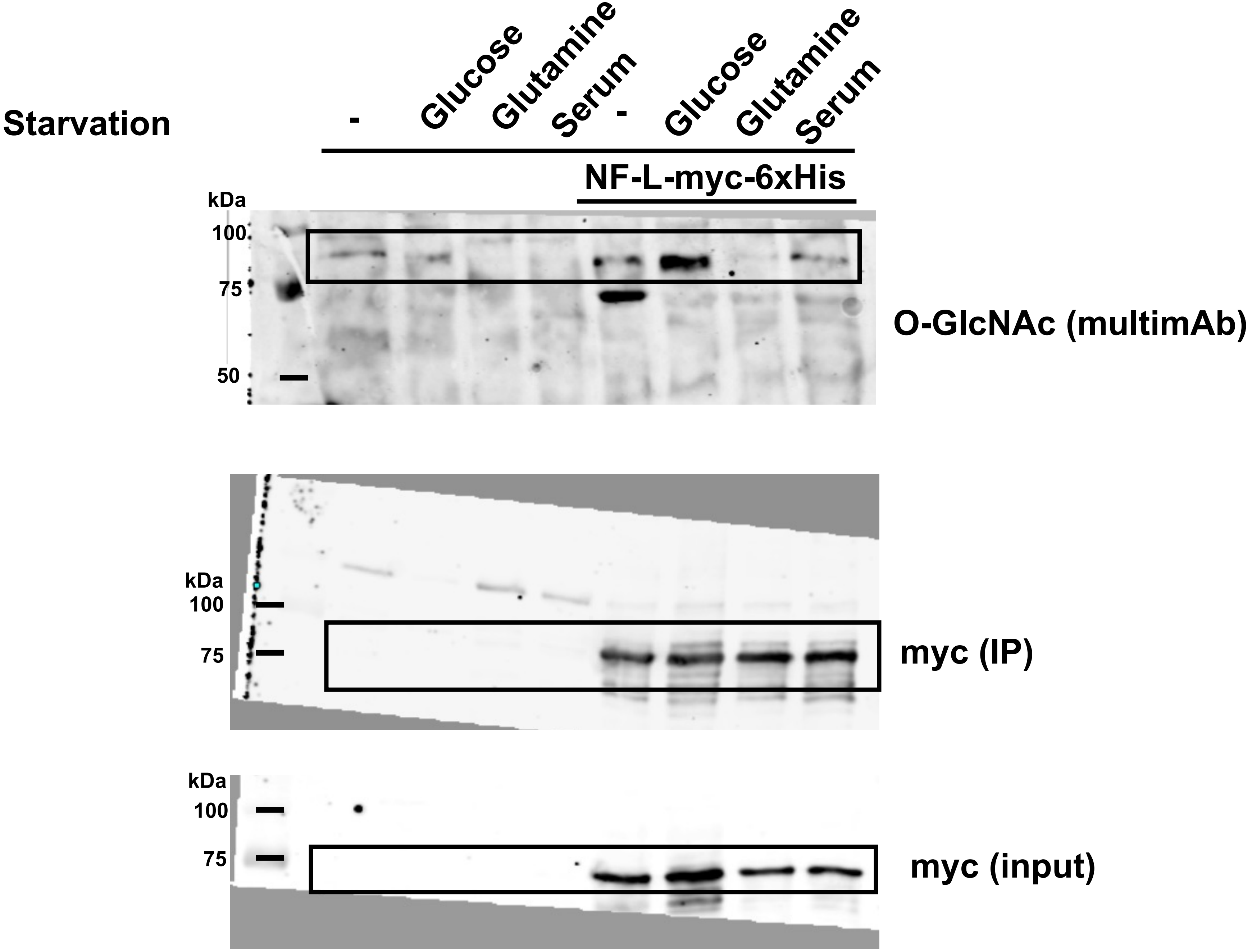
Starvation
-
Glucose
Glutamine
Serum
-
Glucose
Glutamine
Serum
NF-L-myc-6xHis
kDa
100
75
50
O-GlcNAc (multimAb)
kDa
100
75
myc (IP)
kDa
100
75
myc (input)

Figure 5

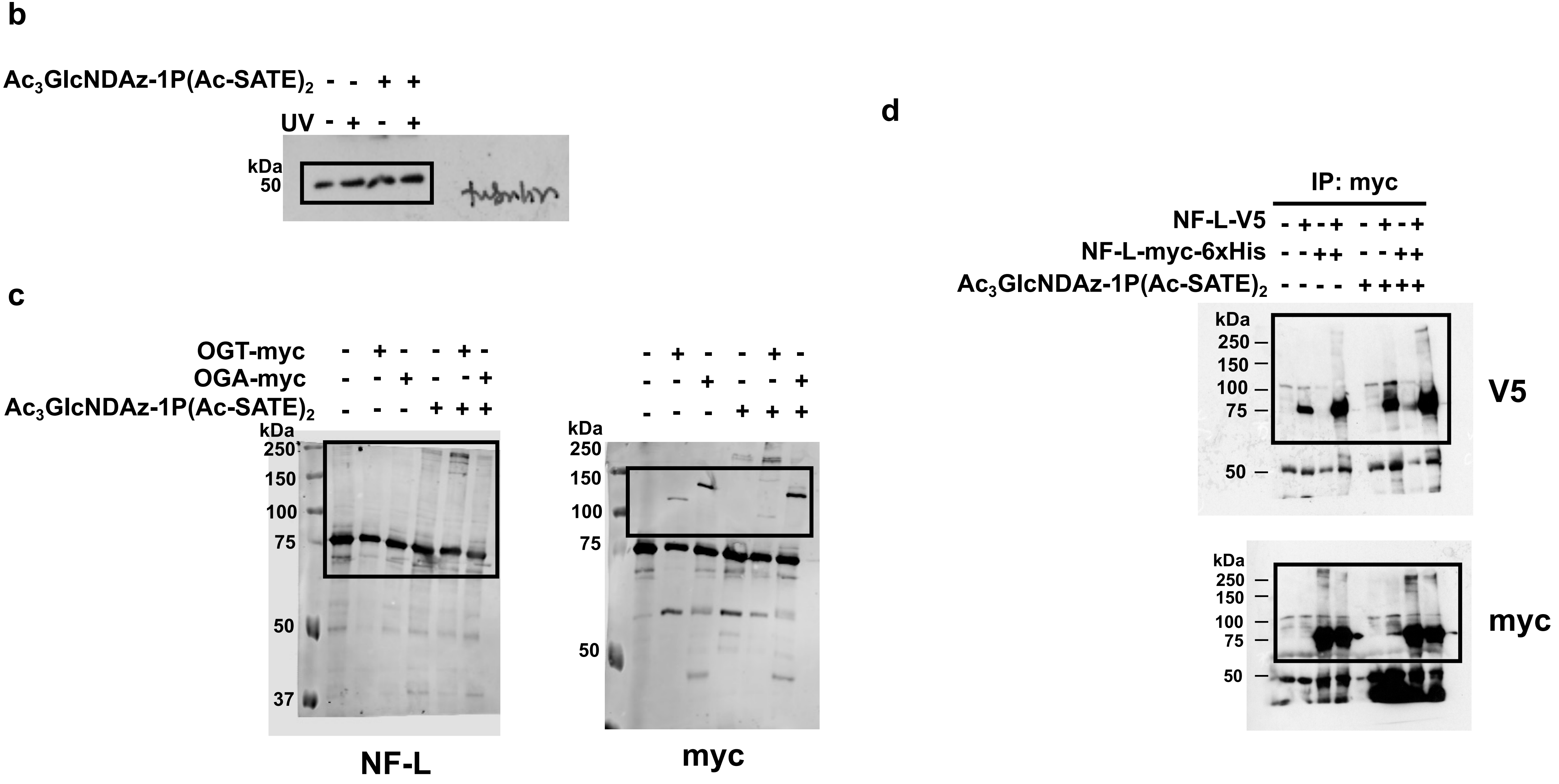

b
Ac3GlcNDAz-1P(Ac-SATE)2 - - + +
UV - + - +
kDa
50
c
OGT-myc
OGA-myc
Ac3GlcNDAz-1P(Ac-SATE)2
kDa
250
150
100
75
50
37
NF-L
myc
d
IP: myc
NF-L-V5
NF-L-myc-6xHis
Ac3GlcNDAz-1P(Ac-SATE)2
V5
myc

Figure 5

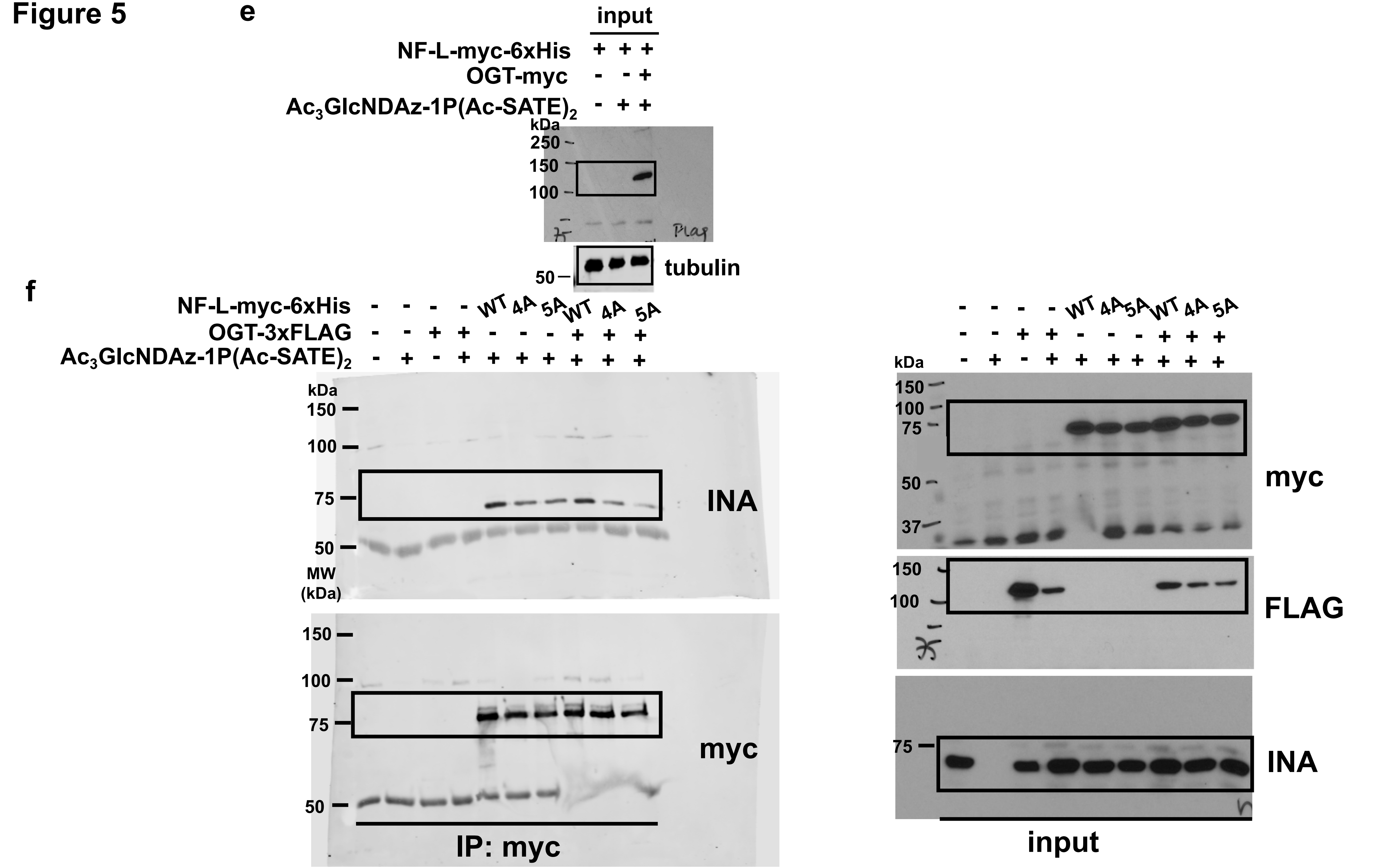
e
input
NF-L-myc-6xHis + + +
OGT-myc - - +
Ac3GlcNDAz-1P(Ac-SATE)2 - + +
kDa
250
150
100
tubulin
50
f
NF-L-myc-6xHis - - - - WT 4A 5A WT 4A 5A
OGT-3xFLAG - - + + - - - + + +
Ac3GlcNDAz-1P(Ac-SATE)2 - + - + + + + + + +
kDa
150
100
75
50
MW (kDa)
INA
myc
IP: myc
37
FLAG
input

Figure 6

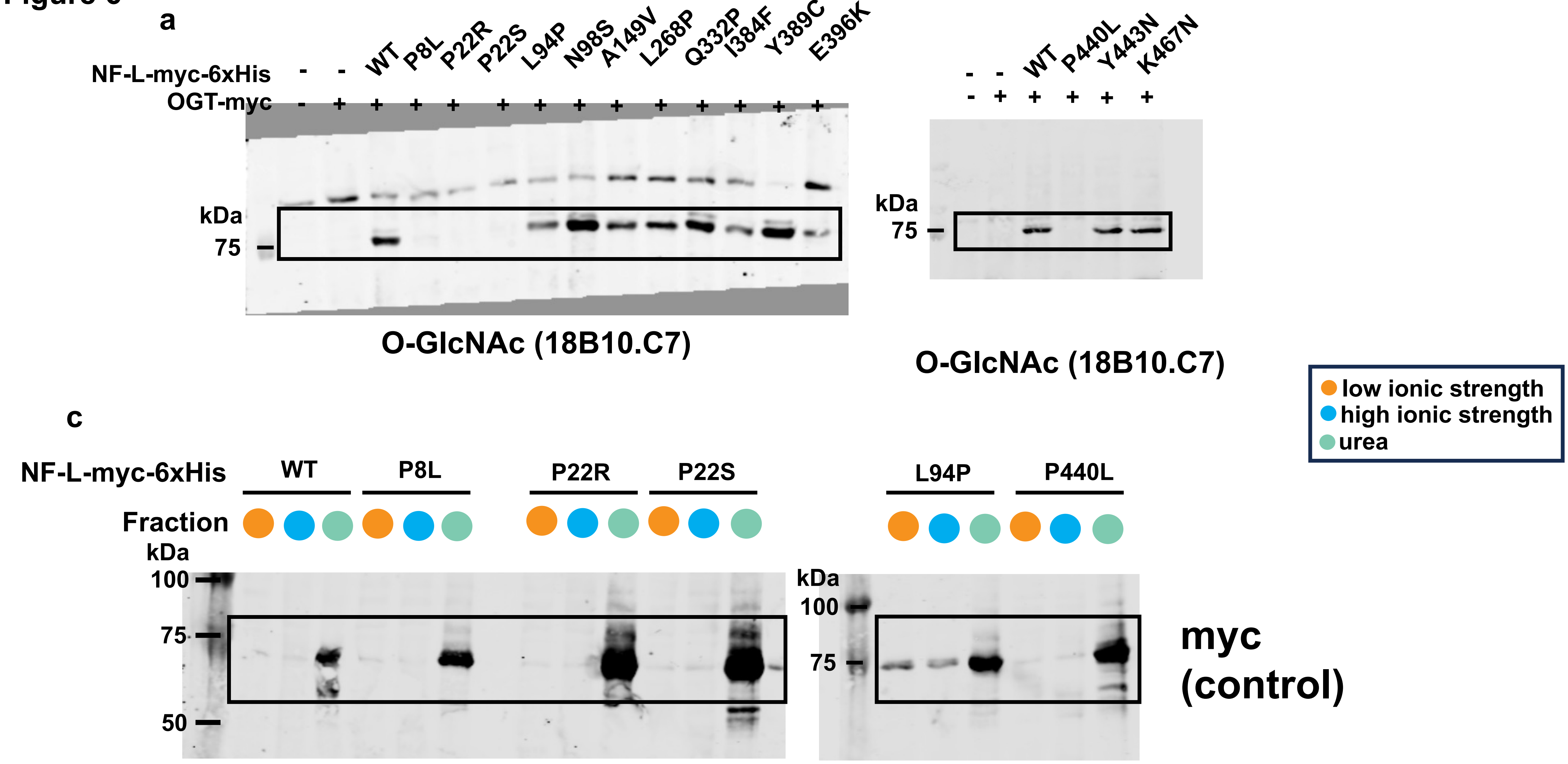
a
NF-L-myc-6xHis - - WT P8L P22R P22S L94P N98S A149V L268P Q332P I384F Y389C E396K
OGT-myc - + + + + + + + + + + + + +
kDa
75
O-GlcNAc (18B10.C7)
- - WT P440L Y443N K467N
- + + + + +
kDa
75
O-GlcNAc (18B10.C7)
low ionic strength
high ionic strength
urea
c
NF-L-myc-6xHis WT P8L P22R P22S L94P P440L
Fraction
kDa
100
75
50
kDa
100
75
myc
(control)

Figure 6

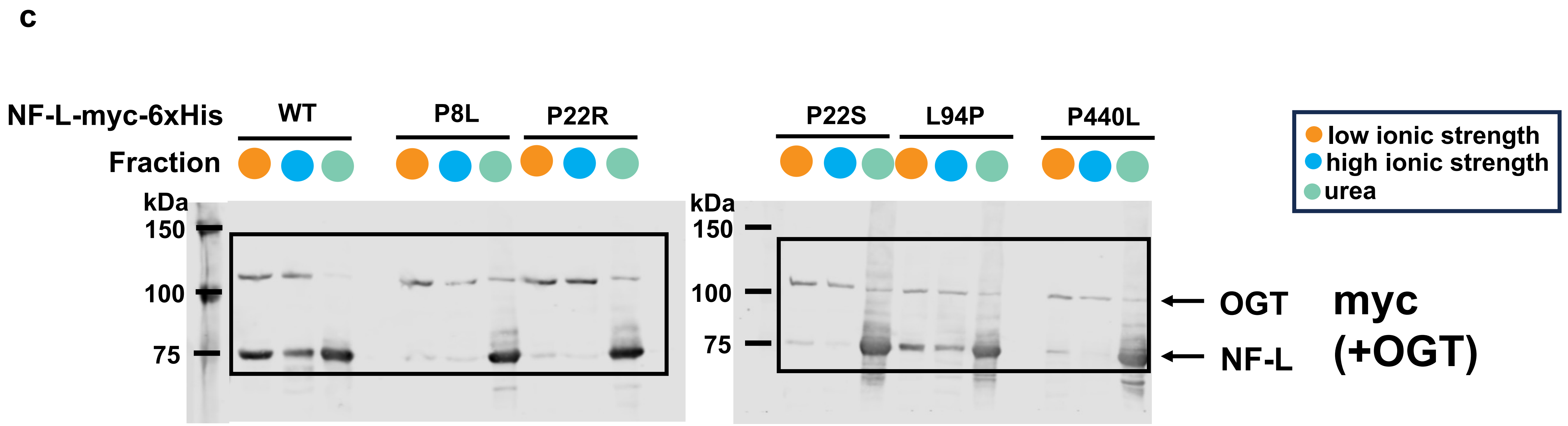
c
NF-L-myc-6xHis
WT
P8L
P22R
P22S
L94P
P440L
Fraction
kDa
150
100
75
kDa
150
100
75
low ionic strength
high ionic strength
urea
OGT
NF-L
myc
(+OGT)

Supplementary Figure 1

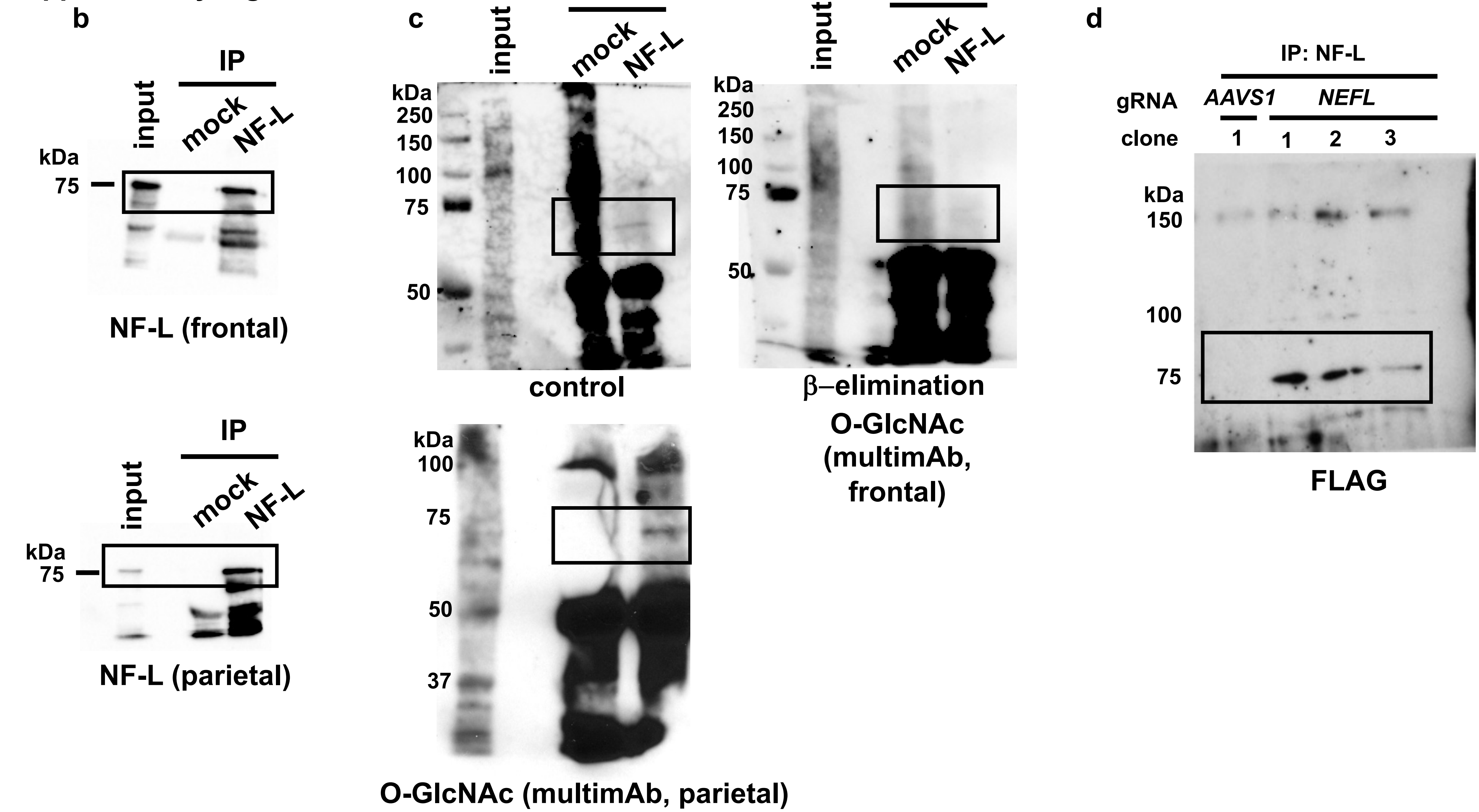

Supplementary Figure 1

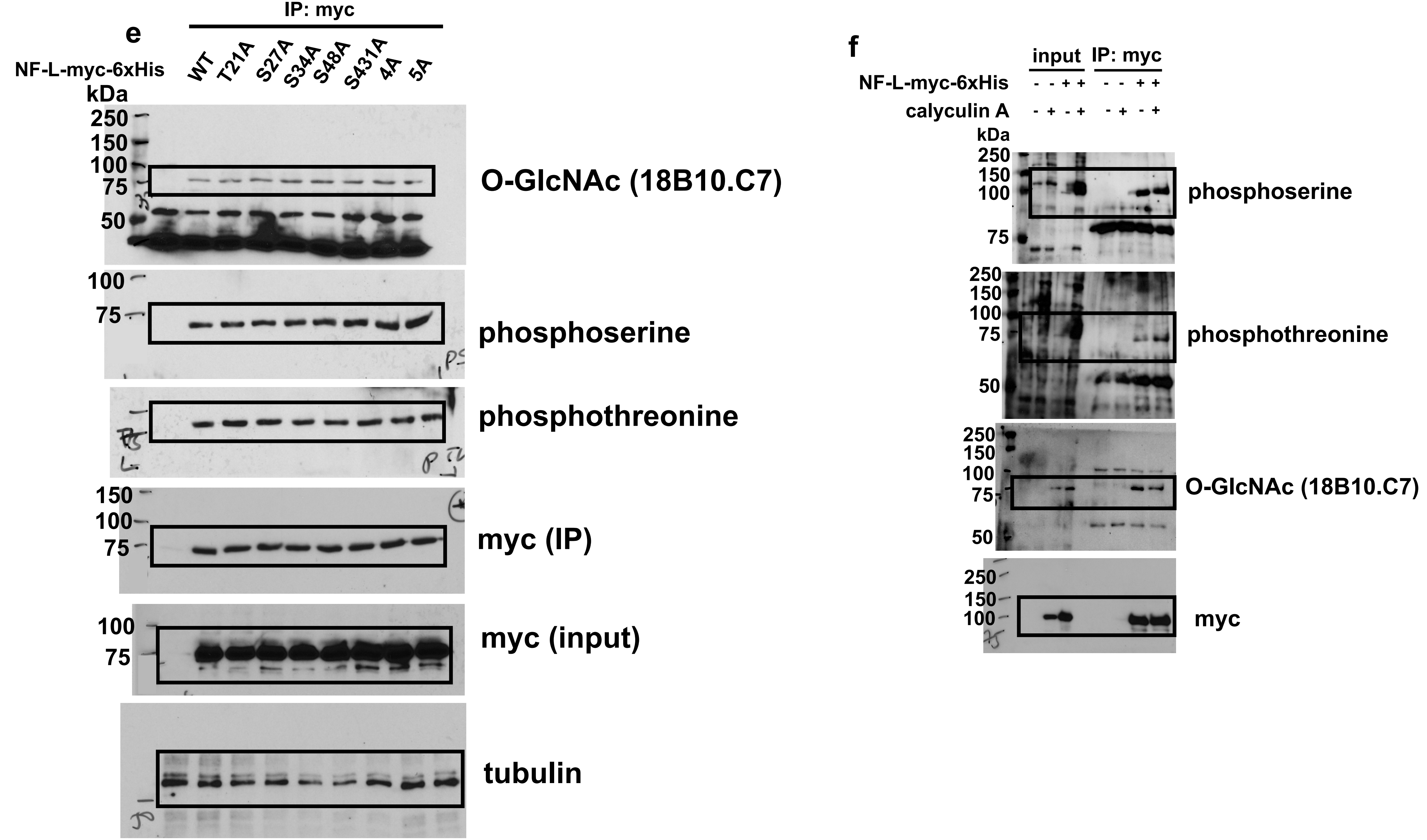

Supplementary Figure 1

g

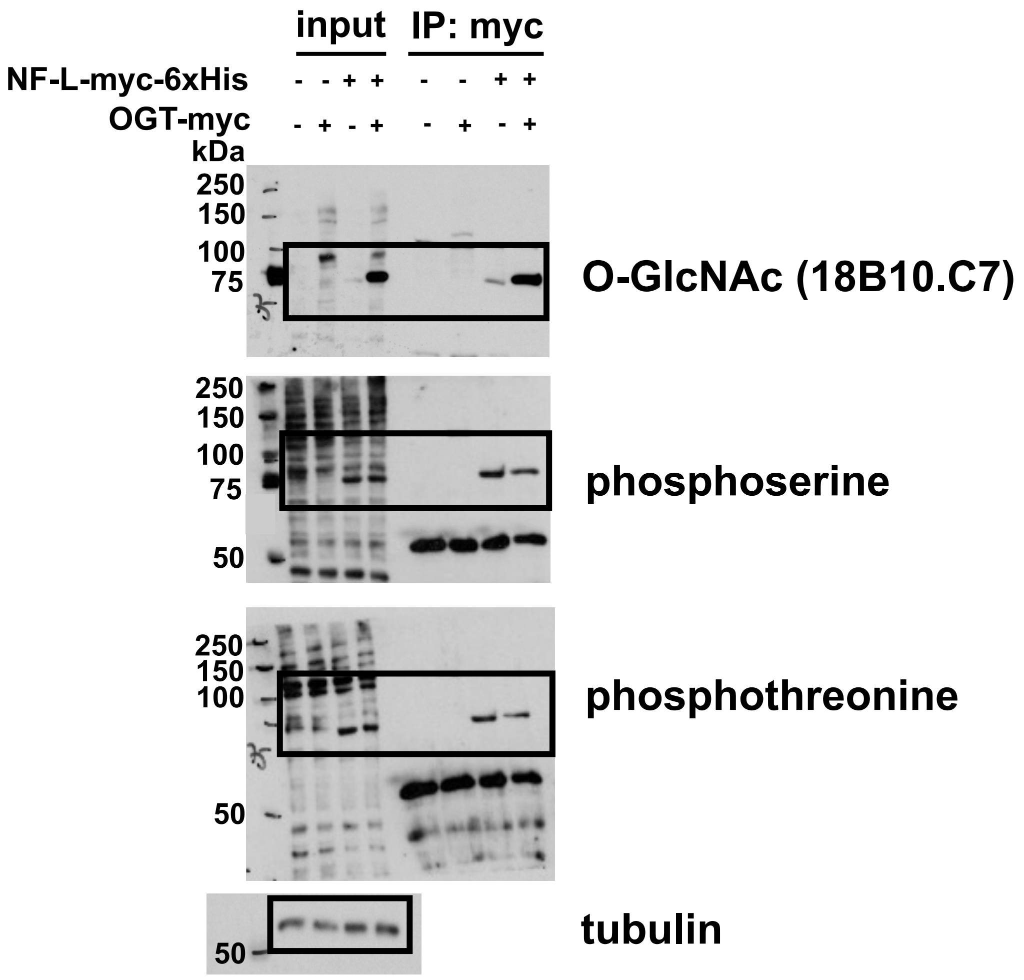

Supplementary Figure 2

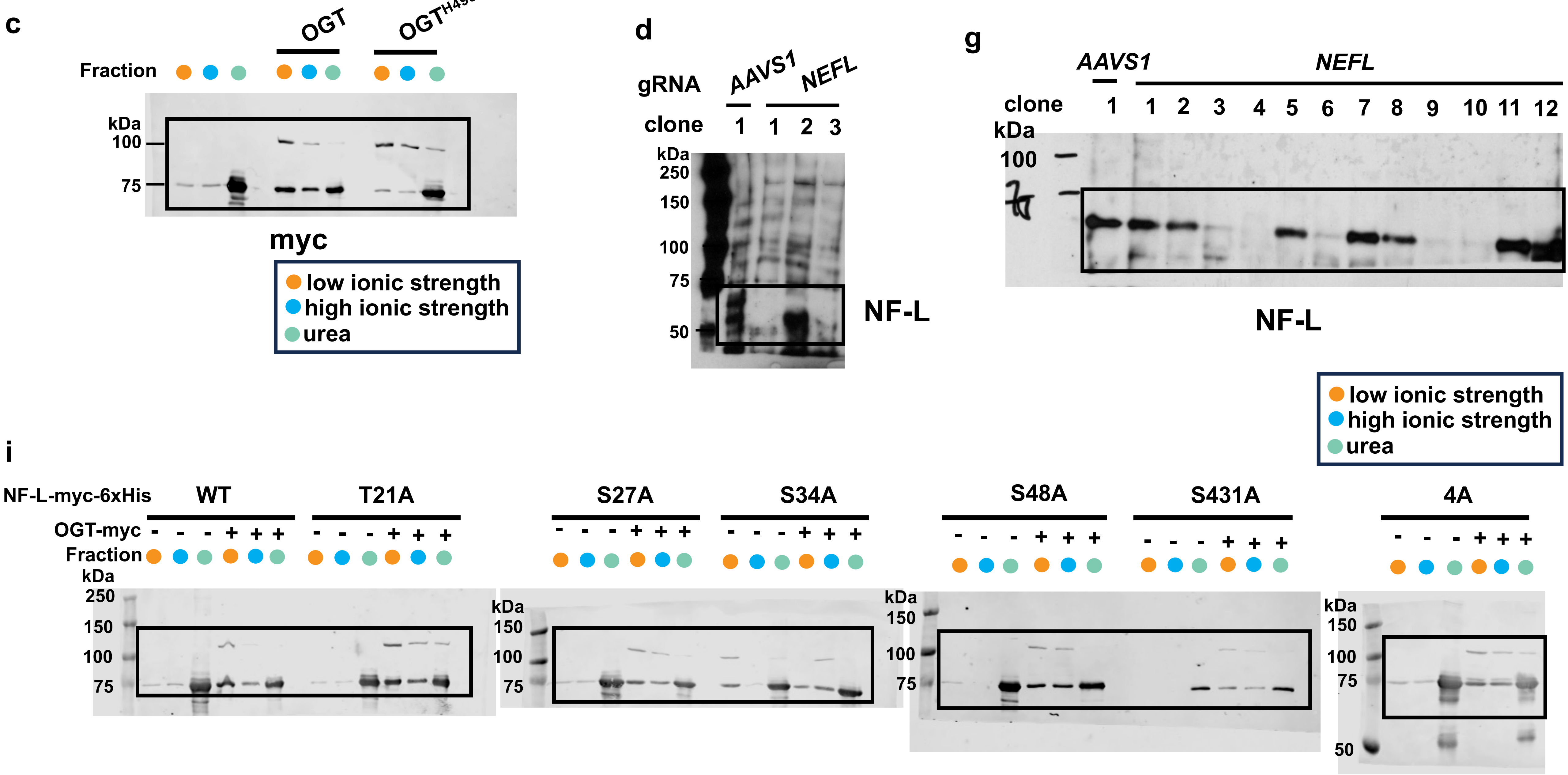

Supplementary Figure 4

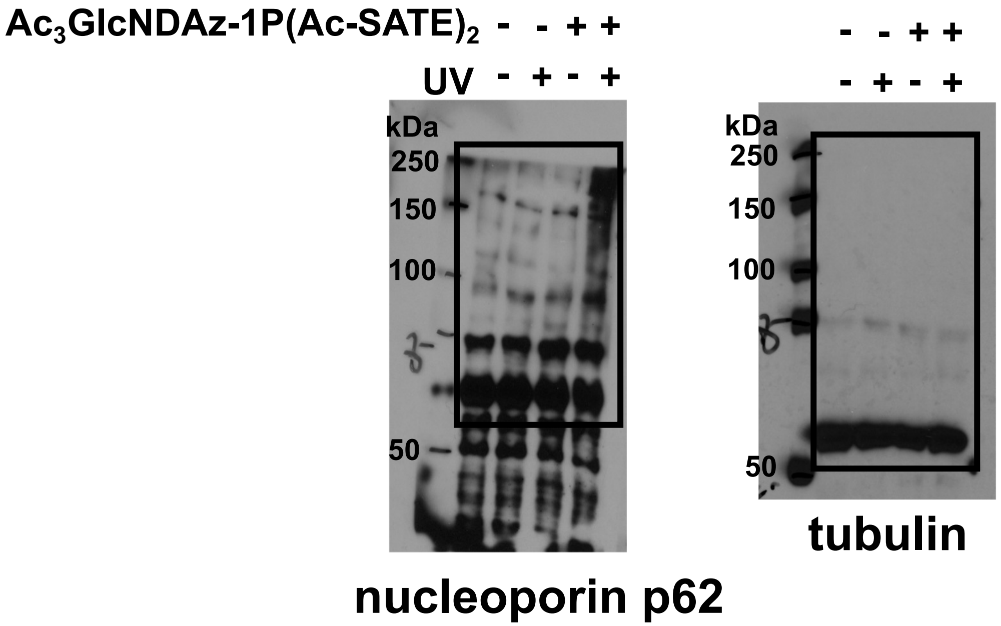

Supplement: Supplementary file 4 — Source Data [file 41467_2023_42227_MOESM4_ESM.zip › NCOMMS-23-09578B Source Data 2.pdf]
